# 

**Published:** 1923-05

**Authors:** 


### Contents

Event and Comment .......... 193
American Dental Association .......... 194
On to Cleveland—September 10-14 .......... 195
Prophylactic Odontotomy: The Cutting Into the Tooth for the Prevention of Disease .......... 196
The Evolution of the Dental Unit .......... 229
General Information Regarding Examination for License to Practice Dentistry in California .......... 231
Immunization Treatment .......... 234
Noblesse Oblige .......... 237
Announcements .......... 239

